# Personalised psychotherapy in primary care: evaluation of data-driven treatment allocation to cognitive–behavioural therapy versus counselling for depression

**DOI:** 10.1192/bjo.2022.628

**Published:** 2023-03-02

**Authors:** Clarissa Bauer-Staeb, Emma Griffith, Julian J. Faraway, Katherine S. Button

**Affiliations:** Department of Psychology, University of Bath, UK; Avon and Wiltshire Mental Health Partnership NHS Trust, UK; Department of Mathematical Sciences, University of Bath, UK

**Keywords:** Depressive disorders, individual psychotherapy, cognitive–behavioural therapies, primary care, outcome studies

## Abstract

**Background:**

Various effective psychotherapies exist for the treatment of depression; however, only approximately half of patients recover after treatment. In efforts to improve clinical outcomes, research has focused on personalised psychotherapy – an attempt to match patients to treatments they are most likely to respond to.

**Aim:**

The present research aimed to evaluate the benefit of a data-driven model to support clinical decision-making in differential treatment allocation to cognitive–behavioural therapy versus counselling for depression.

**Method:**

The present analysis used electronic healthcare records from primary care psychological therapy services for patients receiving cognitive–behavioural therapy (*n* = 14 544) and counselling for depression (*n* = 4725). A linear regression with baseline sociodemographic and clinical characteristics was used to differentially predict post-treatment Patient Health Questionnaire (PHQ-9) scores between the two treatments. The benefit of differential prescription was evaluated in a held-out validation sample.

**Results:**

On average, patients who received their model-indicated optimal treatment saw a greater improvement (by 1.78 PHQ-9 points). This translated into 4–10% more patients achieving clinically meaningful changes. However, for individual patients, the estimated differences in benefits of treatments were small and rarely met the threshold for minimal clinically important differences.

**Conclusion:**

Precision prescription of psychotherapy based on sociodemographic and clinical characteristics is unlikely to produce large benefits for individual patients. However, the benefits may be meaningful from an aggregate public health perspective when applied at scale.

A range of psychotherapies are recommended by the National Institute of Health and Care Excellence for the treatment of depression,^1^ with a large body of evidence that suggesting psychotherapies are equally effective.^2^ However, treatment efficacy remains modest.^3,4^ In the absence of any novel treatments that are clearly superior for everybody, personalised medicine has focused on identifying who responds best to which treatment.^5^ Traditionally, such efforts have been explored with secondary data from randomised controlled trials, which suffer from sample size limitations.^6^ Further methodological limitations include a lack of validation in external samples and the examination of individual characteristics in isolation.^7^ More novel approaches have been developed that take an actuarial approach; these have been implemented in randomised controlled trial data to examine differential treatment effects in depression for cognitive–behavioural therapy (CBT) compared with antidepressant medication,^8^ interpersonal psychotherapy^9^ and psychodynamic therapy.^10^ Furthermore, research has started to use routinely collected data contained in electronic healthcare records. These have the benefit of including much larger patient populations compared with those present in clinical trials. In recent studies, the use of targeted prescription machine learning algorithms to assign patients to CBT versus person-centred counselling for depression (CFD) resulted in approximately 20% greater improvements when patients were assigned to the optimal treatment as indicated by the model.^7^ Further research using a patient profiling algorithm demonstrated that certain patient profiles saw greater improvement in CBT compared with counselling and *vice versa*.^11^ As the implementation of these novel approaches in healthcare records is at a relatively early stage, less is known about the replicability and generalisability of the results. Triangulation of evidence with different methodological approaches and using different samples will add to the evidence base. As such, we used a large-scale sample of healthcare records to assess the benefits of a differential treatment allocation of CBT versus CFD, based on baseline patient characteristics, and to understand which variables contribute to potential differences in clinical outcomes between treatments. The validity of the models was tested in an external data-set.

## Method

### Settings

Improving Access to Psychological Therapies (IAPT) is a national programme that delivers psychological therapy for depression and anxiety across England. IAPT has implemented routine data collection, gathering detailed information about patients, their treatment and their clinical outcomes.^12^ The data are collected on a session-by-session basis to increase complete-case recording, even when patients drop out of treatment early.^12^ The clinical records for the present study were obtained from 15 IAPT services, which were approached based on convenience and feasibility and agreed to participate. The services are located across the south-west of England and London, with the average Index of Multiple Deprivation (IMD) of the sample population ranging from 12.91 to 29.62 among services, the proportion of individuals from a Black, Asian or ethnic minority background ranging from 2.9% to 58.9%, and services being located in a range of settings including both urban inner-city areas and more rural areas. Data from 2012 to late 2019 were included. All data were extracted and fully anonymised using Mayden, the providers of the patient management software used in IAPT, who hold 61% of the market share for adult IAPT services.

### Consent statement

Owing to the anonymous nature of the data, informed consent was neither possible nor required. However, patients who had a record of not wanting their data to be used for further processing were not included in the data extraction.

### Ethics statement

The research received approval from the University of Bath Psychology Research Ethics Committee (19-015).

### Interventions

IAPT operates on a stepped care model, whereby low-intensity therapy (LIT) are offered in the first instance and high-intensity therapy (HIT) is offered where response to LIT is insufficient or where there is a clinical necessity, such as a high baseline severity. CBT and CFD are two of the most commonly available HITs for depression in IAPT. CBT in IAPT is intended to be delivered in accordance with Beck's cognitive model.^13,14^ CFD in IAPT is intended to be delivered as a person-centred, experiential therapy based on the humanistic model.^15^ All therapies are delivered by accredited mental health professionals trained in accordance with the national curriculum.^14,15^

### Sample selection

We identified all patients who had received treatment for clinical levels of depression, based on a diagnosis of depression as well as a depression severity threshold of 10 points on the Patient Health Questionnaire-9 (PHQ-9) at baseline.^16^ Patients were included in the present analysis if the majority HIT they received was CBT or CFD. The majority HIT was defined as the most frequently recorded treatment label within all treatments that fall under the umbrella of HIT in IAPT. LIT was not considered in this definition, but prior LIT was accounted for in the analysis. Patients who received equal amounts of two different HITs were excluded. To allow for pre-and post-treatment measures, patients had to attend at least two appointments. Among patients in this sample, the most recent referral was chosen where patients had a record of multiple prior treatments of CBT or CFD. All patients with missing outcome data at their last attended appointment were excluded. Owing to the session-by-session recording of outcome measures in IAPT, this does not necessarily exclude patients who dropped out of treatment, as their post-treatment score is the measure collected at their last attended appointment. As such, either before dropping out or completing treatment, all patients who completed outcome questionnaires at their last attended session were included.

### Measures

#### Outcome measure

The PHQ-9 is a nine-item self-report questionnaire assessing the severity of depressive symptoms over the past 2 weeks.^16^ Each item is rated on a four-point Likert scale ranging from 0 (‘not at all’) to 3 (‘nearly every day’). The total PHQ-9 score has a range of 0–27, with higher scores indicating greater symptom severity. Scores of 5, 10, 15 and 20 denote mild, moderate, moderately severe and severe depression, respectively. The evidence suggests that a score ≥10 on the PHQ-9 has an 88% sensitivity and specificity for identifying major depressive disorder.

#### Patient characteristics

The baseline variables consisted of data that are routinely collected at the point of referral or assessment. These include sociodemographic data: age, gender, ethnicity, employment status and sexual orientation. We additionally assessed the IMD as a proxy for socioeconomic status.^17^ A range of clinical variables were also collected: disability and long-term health condition status, diagnosis, depression symptoms (PHQ-9), anxiety symptoms (Generalised Anxiety Disorder Scale, GAD-7),^18^ functional impairment (Work and Social Adjustment Scale, WSAS),^19^ psychotropic medication status and referral source. From the available data-set, we determined who had also received LIT and the referral number measuring how many times a patient had been referred to IAPT.

### Statistical analysis

All analyses were performed using the R programming language.^20^

#### Test–training split

Prior to any data analysis, the data-set was randomly split into training and testing samples at a 3:1 ratio to create a held-out validation sample. This has the benefit of allowing the evaluation of the model in a previously unseen data-set. To ensure the training and testing samples are comparable, they must have similar characteristics. As such, the balance of the partitioning was assessed on all variables included in the data analysis using the standardised mean difference (SMD). This included services and referral year. The balance on all variables in the training and test samples was <0.1, meeting a conservative threshold of balance.^21^

#### Imputation

To address missing data, a non-parametric imputation for all baseline characteristics was performed using the ‘missForest’ package, which uses a random forest algorithm.^22^ Random forest imputation has been shown to perform well in data-sets with different data types and outperforms other methods of imputation where there are possible complex interactions and non-linear trends.^22^ As missing outcome data at the last attended appointment was an exclusion criterion, these were not imputed for either training or test data. Random forest imputation was implemented to impute both categorical and continuous variables with 500 trees per forest. Service and year were also included to account for potential differences in patient populations across areas and time. Out-of-bag imputation error estimates were reported to assess the imputation error using the normalised root mean squared error (NRMSE) for continuous variables and the proportion of falsely classified entries (PFC) for categorical variables.^22^ Imputation was performed separately for the training and testing data-sets. Imputation was successful with an NRMSE of 0.40 and a PFC of 0.16.

#### Propensity score estimation

Owing to the observational nature of the data, the allocation to treatment was not random – certain patients may have been more likely to receive one type of treatment over another because of certain characteristics. Propensity scores estimate the probability of receiving one treatment over another based on observed baseline characteristics and can therefore, at least partially, account for patients’ non-random treatment allocation. The propensity scores were added to all subsequent analyses as a covariate in addition to regression adjustment, resulting in a doubly robust approach. Previous research has demonstrated that doubly robust regression adjustment with propensity scores performs well in studies of electronic healthcare records.^23^

#### Treatment model

Arguably, differential treatment allocation is only useful when comparing two equally effective treatments – if one treatment is clearly superior, it would generally be of greater value to simply provide the more effective treatment. Previous research in IAPT suggests that treatment outcomes in CBT and CFD are comparable.^24^ Although the aim of the present analysis was not to evaluate treatment efficacy, in order to assess the equivalence assumption a main effects model was fitted using linear regression, with post-treatment PHQ-9 score as the primary outcome. All baseline patient characteristics and the propensity score were added as covariates. We also adjusted for the total number of appointments to control for treatment dose, the service and the referral year.

#### Prediction model

Non-specific predictors of treatment response are variables that influence how well a patient responds to therapy, irrespective of which treatment they receive, whereas moderators are variables that determine a better or worse response to one treatment over another.^25^ Within statistical models, predictors are coded as main effects and moderators are coded as interactions between a baseline characteristic and treatment. As interactions require more power, a different strategy is to examine specific predictors. Predictors are examined in separate treatment arms to identify which variables are associated with outcomes in a particular treatment, as has been implemented elsewhere.^7^ However, only interactions are able to identify whether variables produce a statistically significant difference in clinical outcomes between treatments. Owing to the larger sample size, we opted to test for interactions.

In the present analysis, a linear regression was fitted in the training data with the patient's post-treatment PHQ-9 score as the primary outcome, covariate-adjusted for baseline PHQ-9.^16,20^ This approach was chosen over change-from-baseline calculations to avoid loss of power and because of the ability to account for measurement error. All baseline characteristics were added into the regression as main effects with an additional interaction term with treatment. We also accounted for the main effects of service, referral year and propensity scores. Likelihood ratio tests were used to assess the significance of predictors and moderators for categorical variables with more than two levels.

To illustrate the magnitude of effect modification, predicted post-treatment PHQ-9 scores were estimated for CBT and CFD separately for each prescriptive variable while keeping the remaining covariates of the model constant. Continuous variables were kept constant at the mean, with categorical variables set to the most frequent level. This effectively allows the moderating effects of baseline characteristics to be isolated. For example, if patient A was female and patient B was male, but they were otherwise identical on all other baseline characteristics, it would be possible to see how much of an impact gender has on clinical outcomes between two different treatments (Table 2).

#### External validation

To test the generalisability of the results, the models were applied to the held-out test set. Within the test data, the post-treatment PHQ-9 score was predicted for CBT and CFD, thereby generating a prediction of the response to the treatment that patients received (a ‘factual’ prediction) as well as for the treatment they did not receive (a ‘counterfactual’ prediction). Following the Personalised Advantage Index (PAI) methodology,^8^ the difference between the two predicted estimates was calculated to define the magnitude of benefit from one treatment over another. This difference quantifies how much better or worse patients would do if they received CBT versus CFD or *vice versa*. The treatment with the lowest predicted PHQ-9 score at the end of treatment is classified as the *optimal* treatment. By contrast, the treatment predicted to produce a higher score is the *suboptimal* treatment.

Previous research has shown that the PAI magnitude is not relevant for all patients – many patients are likely to respond to both treatments similarly.^7,8^ As a means of identifying patients likely to benefit from a differential treatment allocation, we identified patients with a high PAI score. We attempted to identify patients whose PAI exceeded the percent minimal clinically important difference (MCID). This is the smallest difference in scores where patients may experience a subjective improvement, estimated at an approximate reduction of 20% from baseline PHQ-9 scores.^26,27^ However, this number was very small and allowed no meaningful comparison. Previous research has defined a high PAI as a score beyond one standard deviation from the mean.^7^ We adopted a similar approach, defining a high PAI as a score beyond the first or third quartiles, as the distribution was marginally skewed. As such, three groups were defined: patients who received their model-indicated *optimal* treatment, patients who received their model-indicated *suboptimal* treatment and patients where no favourable treatment was indicated by the model.

Subsequently, the observed post-treatment PHQ-9 scores were compared between patients receiving their *optimal* treatment and those receiving their *suboptimal* treatment. This comparison was made for adapted IAPT metrics of recovery, reliable change and reliable recovery.^28^ Recovery was defined as falling above clinical cut-offs on either depression or anxiety questionnaires pre-treatment and falling below these clinical cut-offs on depression and anxiety post-treatment.^28^ The depression measure used in IAPT is the PHQ-9, and the clinical cut-off is ≥10 points.^28^ Reliable change is measured as pre- and post-treatment questionnaire changes exceeding the measurement error on one or both depression or anxiety questionnaires (without a reliable deterioration on the other).^28^ The reliable change threshold on the PHQ-9 is ≥6 points.^28^ Reliable recovery is defined as a change in scores that exceeds the measurement error and scores falling below the clinical cut-offs.^28^ In the present study these definitions were adapted to only incorporate the depression measure rather than a combination of depression and anxiety measures, as the primary interest in the present study was depression. Furthermore, the comparison was also made for both a percent MCID, which is defined as a 20% reduction from baseline, and an absolute MCID, which has a range of values specific to baseline severity.^26,27,29^ The difference/odds ratio of the outcomes between patients who received their optimal and suboptimal treatments were determined using simple linear and logistic regression, respectively.

## Results

### Sample characteristics

The majority of the sample were women (67%) and White (79.9%), with an average age of 40 years. Most patients had moderately severe depression (18 PHQ-9 points), moderate anxiety (14 GAD-7 points) and moderately severe functional impairment (23 WSAS points). There were differences in baseline characteristics in patients receiving CBT and CFD. Sample characteristics are described in Table 1, with an SMD threshold of <0.25 indicating adequate balance.^20^ Patients who received CBT appeared to be more likely to have a diagnosis of recurrent depressive disorder, higher levels of depressive symptoms and greater functional impairment. They also appeared to be more likely to be taking psychotropic medication and more likely to have self-referred and to have received LIT, as well as having a higher referral number. This difference in baseline characteristics potentially suggests that patients are already being allocated to treatment based on their clinical profile. However, it should be noted that these imbalances are not adjusted for other variables. As such, they could be a consequence of specific services having different populations and delivering a different ratio of CBT to CFD.

**Table 1:** Baseline characteristics of patients, stratified by treatment

		Total sample	Cognitive–behavioural therapy	Counselling for depression	Standardised mean difference
*N*		19 269	14 544	4725	
Age		40.25 (13.99)	39.59 (14.01)	42.31 (13.72)	0.196
Gender	Female	12 837 (66.6)	9479 (65.2)	3358 (71.1)	0.127
	Male	6432 (33.4)	5065 (34.8)	1367 (28.9)	
Ethnicity	White	15 401 (79.9)	11 983 (82.4)	3418 (72.3)	0.242
	Black, Asian or ethnic minority	3868 (20.1)	2561 (17.6)	1307 (27.7)	
Employment Status	Employed	10 570 (54.9)	7766 (53.4)	2804 (59.3)	0.120
	Not working	8699 (45.1)	6778 (46.6)	1921 (40.7)	
Index of Multiple Deprivation		21.47 (11.85)	21.55 (12.12)	21.23 (10.99)	0.027
Sexual orientation	Heterosexual	18 366 (95.3)	13 816 (95.0)	4550 (96.3)	0.064
	Not heterosexual	903 (4.7)	728 (5.0)	175 (3.7)	
Disability	No	16 449 (85.4)	12 415 (85.4)	4034 (85.4)	<0.001
	Yes	2820 (14.6)	2129 (14.6)	691 (14.6)	
Long-term health condition	No	12 602 (65.4)	9448 (65.0)	3154 (66.8)	0.038
	Yes	6667 (34.6)	5096 (35.0)	1571 (33.2)	
Diagnosis	Depressive episode	14 325 (74.3)	10 288 (70.7)	4037 (85.4)	0.361
	Recurrent depressive disorder	4944 (25.7)	4256 (29.3)	688 (14.6)	
Baseline PHQ-9		18.24 (4.39)	18.52 (4.35)	17.40 (4.39)	0.255
Baseline GAD-7		14.34 (4.52)	14.52 (4.49)	13.80 (4.58)	0.159
Baseline WSAS		23.07 (8.56)	23.76 (8.38)	20.94 (8.76)	0.329
Psychotropic medication	Yes	11 112 (57.7)	8879 (61.0)	2233 (47.3)	0.279
	No	8157 (42.3)	5665 (39.0)	2492 (52.7)	
Referral source	Self	10 669 (55.4)	8607 (59.2)	2062 (43.6)	0.432
	Primary care	7373 (38.3)	4850 (33.3)	2523 (53.4)	
	Other	1227 (6.4)	1087 (7.5)	140 (3.0)	
Referral number		1.79 (1.25)	1.86 (1.31)	1.56 (1.00)	0.252
Low-intensity therapy	No	14 007 (72.7)	10 169 (69.9)	3838 (81.2)	0.266
	Yes	5262 (27.3)	4375 (30.1)	887 (18.8)	

PHQ-9, Patient Health Questionnaire (nine-item); GAD-7, Generalised Anxiety Disorder Scale (seven-item); WSAS, Work and Social Adjustment Scale. Continuous data are presented as mean (standard deviation) and categorical data are presented as *n* (%).

### Main treatment effects

We found no evidence to suggest there are significant differences in treatment outcomes between CBT versus CFD in this sample within a main effects model. After adjusting for baseline and treatment characteristics, the difference in post-treatment PHQ-9 score between treatments was −0.10 (95% CI −0.39 to 0.18, *P* = 0.493).

### Non-specific predictors and moderators of treatment outcomes

Lower age, not working, higher IMD, having a disability or long-term health condition, and higher baseline PHQ-9, GAD-7 and WSAS scores were predictors of higher post-treatment PHQ-9 scores across both CBT and CFD (see Supplementary Table C3 available at https://doi.org/10.1192/bjo.2022.628). Furthermore, taking medication, being referred from primary care or other services (versus self-referring) and having a higher referral number were identified as predictors of worse outcomes. Service and year were also predictive of clinical outcomes. After adjusting for other baseline characteristics, we found no evidence to suggest that gender, ethnicity, sexual orientation or also receiving LIT was associated with outcomes.

We found weak evidence that employment status and psychotropic medication were moderators of clinical outcomes in CBT versus CFD, but differences were of a very small clinical magnitude when other covariates were kept constant (Table 2). Other moderators also appeared to make small differences in clinical outcomes when other covariates were kept constant, but none of these differences reached statistical significance.

**Table 2:** Illustration of moderating effects for baseline characteristics on predicted post-treatment PHQ-9 scores in cognitive–behavioural therapy versus counselling for depression with other covariates held constant

	Cognitive–behavioural therapy		Counselling for depression		
		Beta	95% CI	Beta	95% CI	*P-*value
Age	28	9.50	9.06−9.94	9.56	8.90−10.21	0.116
	50	8.70	8.28−9.11	9.10	8.47−9.73	
Gender	Female	9.05	8.64−9.46	9.30	8.69−9.91	0.679
	Male	9.16	8.71−9.61	9.52	8.82−10.22	
Ethnicity	White	9.05	8.64−9.46	9.30	8.69−9.91	0.205
	Black, Asian, and minority ethnic	9.39	8.86−9.92	10.02	9.27−10.77	
Employment status	Employed	9.05	8.64−9.46	9.30	8.69−9.91	0.050
	Not working	10.97	10.52−11.42	10.69	10.00−11.37	
Index of Multiple Deprivation	12	8.81	8.39−9.23	9.18	8.55−9.81	0.237
	29	9.25	8.83−9.67	9.39	8.76−10.02	
Sexual orientation	Heterosexual	9.05	8.64−9.46	9.30	8.69−9.91	0.262
	Not heterosexual	9.56	8.88−10.23	9.08	7.78−10.37	
Disability	No	9.05	8.64−9.46	9.30	8.69−9.91	0.924
	Yes	9.73	9.18−10.28	9.94	9.06−10.82	
Long-term condition	No	9.05	8.64−9.46	9.30	8.69−9.91	0.603
	Yes	9.62	9.17−10.06	10.01	9.32−10.70	
Diagnosis	Depressive episode	9.05	8.64−9.46	9.30	8.69−9.91	0.686
	Recurrent depressive disorder	9.35	8.85−9.85	9.46	8.63−10.29	
Baseline PHQ-9	15	7.87	7.45−8.29	8.20	7.57−8.84	0.466
	22	10.43	9.99−10.87	10.57	9.91−11.24	
Baseline GAD-7	11	8.62	8.19−9.04	8.86	8.23−9.50	0.982
	18	9.53	9.10−9.96	9.78	9.13−10.42	
Baseline WSAS	17	8.60	8.18−9.02	8.92	8.30−9.54	0.463
	30	9.57	9.13−10.00	9.73	9.08−10.38	
Psychotropic medication	Yes	9.05	8.64−9.46	9.30	8.69−9.91	0.049
	No	8.59	8.18−9.00	8.32	7.72−8.92	
Referral source	Self	9.05	8.64−9.46	9.30	8.69−9.91	0.211
	Primary care	9.71	9.24−10.18	9.58	8.96−10.21	
	Other	10.02	9.42−10.63	10.83	9.48−12.18	
Referral number	1	8.81	8.39−9.22	9.05	8.42−9.68	0.983
	2	9.12	8.71−9.53	9.37	8.76−9.98	
Low-intensity therapy	No	9.05	8.64−9.46	9.30	8.69−9.91	0.205
	Yes	9.24	8.71−9.76	9.88	9.07−10.70	

PHQ-9, Patient Health Questionnaire (nine-item); GAD-7, Generalised Anxiety Disorder Scale (seven-item); WSAS, Work and Social Adjustment Scale. For each characteristic, the treatment and one moderator are varied while all other baseline characteristics are held constant at the mean for continuous variables or most common level for categorical variables to illustrate the magnitude of effect modification.

### External cross-validation

The discrepancy between the actual post-treatment score and the model predicted score was −0.19 (s.d. = 6.44). The median PAI in the test sample was 0.11 (interquartile range: −0.29 to 0.53). This suggests that across all patients in the test sample, more patients may marginally benefit from CFD. However, as was found in previous research, these small differences suggest that not all patients benefit from a differential treatment allocation. As such, we identified patients who may benefit the most by selecting those with a PAI beyond the first and third quartiles. In this 50% of patients, 1247 (51.8%) received their model-indicated optimal treatment. Where the model indicated CBT as the optimal treatment, i.e. where according to the model offering CBT would be favourable, 944 (78.3%) of patients received CBT. Where CFD was the model-indicated optimal treatment, i.e. where according to the model offering CFD would be more beneficial, 303 (25.2%) of patients received CFD.

Patients who received their optimal treatment scored −1.78 (95% CI −2.36 to −1.21, P < 0.001) PHQ-9 points lower than those who received their suboptimal treatment (Table 3). Patients in the optimal group had a mean post-treatment PHQ-9 score of 9.63 (s.d. = 6.95), whereas the suboptimal group scored 11.42 (s.d. = 7.49). The odds of recovery for those receiving their optimal treatment versus those who received their suboptimal treatment was 1.52 (95% CI 1.29 to 1.79, P < 0.001); 60.0% of patients in the optimal group recovered compared with 49.7% in the suboptimal group. The odds of achieving a reliable change for those receiving their optimal treatment versus those who received their suboptimal treatment was 1.19 (95% CI 1.01 to 1.40, P = 0.038); 63.8% of patients in the optimal group recovered compared with 59.7% in the suboptimal group. The odds of achieving a reliable recovery for those receiving their optimal treatment versus those who received their suboptimal treatment was 1.35 (95% CI 1.15 to 1.58, P < 0.001); 53.9% of patients in the optimal group recovered compared with 46.5% in the suboptimal group. The odds of achieving a percent MCID of a 20% improvement from baseline for those receiving their optimal treatment versus those who received their suboptimal treatment was 1.37 (95% CI 1.15 to 1.64 *P* < 0.001) with 74.2% of patients in the optimal group showing changes of a clinically meaningful magnitude compared with 67.6% in the suboptimal group. The odds of achieving an absolute MCID for those receiving their optimal treatment versus those who received their suboptimal treatment was 1.39 (95% CI 1.18 to 1.63 P < 0.001); 63.8% of patients in the optimal group recovered compared with 56.0% in the suboptimal group.

**Table 3:** Evaluation of a data-driven treatment allocation model in held-out test sample

	Optimal treatment	Suboptimal treatment	Beta/odds ratio	95% CI	*P*-value
*N*	1247	1162			
Post-treatment PHQ-9	9.63 (6.95)	11.42 (7.49)	−1.78	−2.36 to −1.21	<0.001
Recovery	748 (60.0)	577 (49.7)	1.52	1.29−1.79	<0.001
Reliable change	796 (63.8)	694 (59.7)	1.19	1.01−1.40	0.038
Reliable recovery	672 (53.9)	540 (46.5)	1.35	1.15−1.58	<0.001
MCID (%)	925 (74.2)	786 (67.6)	1.37	1.15−1.64	<0.001
MCID (absolute)	796 (63.8)	651 (56.0)	1.39	1.18−1.63	<0.001

PHQ-9, Patient Health Questionnaire (nine-item); MCID, minimal clinically important difference.

When exploring the baseline characteristics of patients who were predicted to have better treatment responses in CBT, we found that they tended to be slightly older and have lower IMD and depression, anxiety and functional impairment scores. This group also had a higher proportion of patients who self-referred, were employed and were heterosexual. Furthermore, better response to CBT was predicted among those who received LIT prior to HIT, had a long-term health condition and were taking medication, relative to the CFD group. Conversely, patients who were predicted to have better treatment responses in CFD tended to be slightly younger, as well as having higher IMD and depression, anxiety and functional impairment scores. This group also had a higher proportion of patients who were referred from primary care, were not working, were not heterosexual and had no previous LIT. Furthermore, it had a higher proportion of patients who were not taking medication and had no long-term health conditions. Proportions of gender, ethnicity, disability status, diagnosis and referral number appeared to be similar.

## Discussion

Electronic healthcare records were used to identify a cohort of patients receiving CBT or CFD for depressive symptoms in primary care settings. We investigated the benefit of differential treatment allocation on the basis of baseline characteristics. The results were validated in a held-out test sample. We found no evidence to suggest a main effect of treatment for CBT or CFD. However, we found some evidence to suggest that differential treatment allocation based on baseline characteristics could modestly improve outcomes. When allocated to their model-indicated optimal treatment, patients improved 1.8 points more on the PHQ-9 compared with patients who were allocated to their suboptimal treatment. This resulted in 4–10% more patients achieving favourable clinical outcomes. However, there were very few patients for whom the predicted difference between treatments was of a clinically meaningful magnitude at the individual level. However, benefits may nonetheless be meaningful from a public health perspective when applied at the population level.

### Discussion of findings

Similar to previous research, which compared CBT and counselling, we found no evidence of a main treatment effect of CBT versus CFD in patients with depression.^24^ However, previous research has shown that some patients can benefit if they are differentially allocated to CBT versus CFD on the basis of baseline characteristics.^7^ Previous research used a supervised machine learning algorithm to identify predictors separately within each treatment.^7^ This approach of examining predictors separately in each treatment group is favourable, relative to testing for interactions, when sample sizes are smaller as there is insufficient power to assess moderating effects (i.e. to test for interactions). The present study tested for moderation in a larger sample, which has the benefit of additional power to assess differential effects of characteristics in different treatments. In the previous research study, 62.5% of patients experienced a reliable recovery if they were assigned to their optimal treatment, whereas only 41.7% of patients achieved this if they were assigned to their suboptimal treatment (among the 30% of people who benefited from a differential treatment allocation).^7^ This approximately 20% difference in improvement translated into post-treatment PHQ-9 differences in the range of approximately 1–2 points and effect sizes ranging from 0.16 to 0.33.^7^ We found comparable benefits on the post-treatment PHQ-9 but much more modest improvements in reliable recovery. It has been suggested that higher deprivation may be associated with worse outcomes of CBT and better outcomes of CFD.^7^ Ethnicity was found to only be a predictor for CBT, with ethnic minority groups having worse outcomes.^7^ Higher baseline anxiety, lower outcome expectancy, longer chronicity and not taking antidepressant medication were found to be associated with better outcomes in CFD only.^7^ Our research suggests that only two variables were marginally statistically significant moderators. Similar to the previous study, we found some evidence to suggest that medication status is a moderator; however, contrary to previous research, we also found that employment status was a moderator, whereas this was found to be a general predictor in the previous study.^7^ In our work, no other variable reached statistical significance when testing for effect modification. However, owing to the previous study including additional variables, only crude comparisons of variables can be made. Further research used a patient profiling algorithm to identify distinct groups of patients with specific profiles and examined differences in treatment response. Certain patient profiles showed greater clinical improvements in CBT, whereas other patient profiles appeared to benefit more from counselling, although the point estimates for the latter groups had wider confidence intervals.^11^

We found no substantial evidence to support the idea that any of the examined moderators produce meaningfully different clinical outcomes individually. Perhaps surprisingly, we still observed benefits between patients who received their optimal versus suboptimal treatment at a group level. This potentially suggests that no individual characteristic is sufficient to result in substantive effect modification; rather, there may be a cumulative effect – small differences may add up across multiple characteristics. It should be noted that the benefits were only observed at the population level – almost no patients had a PAI score that reached the threshold of an MCID.^26,27,29^ This suggests that benefits may not be immediately tangible to every individual patient; rather, they appear to be relevant from a public health perspective, with clinical outcomes improved to a small degree but at scale. However, it should be noted that achieving a difference beyond the MCID at the individual level, a reduction of approximately 20% from baseline, may be a relatively ambitious threshold given that both treatments are generally effective.^26,27^

### Strengths and limitations

The present study used a large, retrospective cohort of patients receiving treatment for depression in primary care, from multiple services across different geographic locations. This, in addition to the naturalistic settings, increases the external validity and generalisability of the findings. Furthermore, we used pre–post treatment outcome measures, which are favourable to retain power and account for measurement error. We also validated the model in an external test sample.

Despite the large, diverse sample, it is still possible that the heterogeneity which exists between services may nonetheless limit the generalisability to other services.^30^ A further limitation of the present research is its observational nature. Unlike in randomised controlled trials, patients in routine clinical practice are not randomly allocated to treatments. We found differences in the baseline characteristics of patients between treatments, which may suggest that patients with a higher clinical severity were more likely to receive CBT. We applied doubly robust propensity adjustment, which has been established as performing well in electronic healthcare records.^23^ However, adjustment can only be made for observed variables, leaving the possibility of unmeasured confounding. Possible examples may include, but are not limited to, mental health comorbidities,^31^ childhood maltreatment,^32^ cognitive biases,^33^ competency in cognitive skills^34^ and shame.^35^ In addition, whereas all treatments in IAPT are delivered by mental health professionals, who are trained in accordance with the national curriculum, there are currently no measures of treatment fidelity in IAPT, making judgements about the adherence to treatment protocols difficult.^36^ As such, the present study serves as an explanatory exploration, with more rigorous and causal research required prior to application in practice, such as evaluations in a randomised controlled trial.

Furthermore, the present results are limited by the data quality of routinely collected data. Electronic healthcare records contain missing data and are collected by various clinicians across different services and years. We used robust methods of data imputation to address missingness but were unable to account for any systemic differences in data recording by individual therapists and/or services. We examined broad sociodemographic and clinical characteristics. Previous research has identified more detailed psychological characteristics including cognitive problems, attributional style, and interpersonal self-sacrificing that may have moderating effects.^9^ These more in-depth psychological characteristics may be promising moderators that produce greater differential improvements at the patient level, but we were limited by data availability. Last, a pragmatic limitation was that the present research did not take organisational factors into account, such as treatment availability. Given the small benefit at the patient level, therapy that is available immediately may outweigh the benefit of waiting for the model-indicated treatment. Such decisions would need to be weighed by the treating clinician.

### Implications

The present approach could potentially inform clinical decision-making. Although the benefits of differential treatment allocation may not produce large effects, and no characteristics emerged as strong moderators at the patient level, they may still be relevant from a public health perspective when applied at scale. Currently, IAPT services in England receive approximately 1.7 million referrals per year.^4^ As such, improvements in clinical outcomes ranging from approximately 4–10% may still affect a large number of patients. However, only randomised controlled trials can determine the true extent of the benefits of differential treatment allocation based on baseline characteristics. A benefit of the present approach is that it comes at minimal cost and is easy to implement, resulting in little burden to healthcare systems. In addition, there is little risk concerning the implementation, as patients receive one of two effective treatments.

Beyond the immediate implications, the present research touches on a debate in the current literature concerning the mechanisms by which therapy produces change. The debate focuses on whether these mechanisms are common and shared across therapeutic modalities or whether there are specific factors unique to different approaches.^37^ The identification of differential outcomes of CBT versus CFD based on baseline characteristics potentially suggests that each may possess specific factors; however, the effects we found were modest. Furthermore, the finding that treatments appear to be equally effective and that most characteristics are stronger general predictors of response, rather than moderators, also speaks to the idea that various common factors are likely to exist. Our research suggests that common factors are likely to contribute to outcomes, but that specific factors may also contribute to a small yet potentially clinically relevant degree.^37^

In order to have greater confidence in differential treatment allocation, an understanding of the mechanisms of depression as well as how treatments work is necessary. However, no clear consensus has yet been established in process research that elucidates the mechanism of action underpinning psychotherapy.^38^ This is further complicated by the fact that the evidence for mechanisms of depression remains unclear, as well as the complexity of depression as evidenced by the significant symptom heterogeneity.^39^ This makes it challenging to reconcile depressive and therapeutic mechanisms and moderator research to assess whether they converge, at the very least on a theoretical basis. Future research investigating the mechanisms of both psychotherapy and psychopathology will undoubtedly provide invaluable insights that could guide efforts to match patients to their optimal treatment.

### Future prospects

The present research suggests that targeted allocation of psychotherapy based on baseline characteristics has the potential to personalise therapy, but only to some degree. Although the effects were modest at the patient level, the impact from a public health perspective may nonetheless be meaningful. Owing to their ease of implementation, minimal risk and low cost, such models provide a simple way to support clinicians in clinical decision-making in the future. However, causal research is necessary to truly evaluate the benefit of personalised approaches to the treatment of depression. Furthermore, significant advances in personalised psychotherapy are likely to depend on advances in the mechanistic understanding of psychopathology itself, as well as how psychotherapy works, in order to optimally match treatments to disease-specific processes.

## Data Availability

The present study contains anonymous, individual-level secondary data in the form of electronic healthcare records from IAPT services. Data ownership lies with the National Health Service (NHS). As such, the data cannot be made available by the authors. Access to individual-level data from the NHS requires an application through the Health Research Authority.

## Supplementary material

For supplementary material accompanying this paper visit http://doi.org/10.1192/bjo.2022.628. click here to view supplementary material
