# Supplementary material for: Personalised psychotherapy in primary care: evaluation of data-driven treatment allocation to cognitive–behavioural therapy versus counselling for depression
Source: BJPsych Open. 2023 Mar 2;9(2):e46. doi: 10.1192/bjo.2022.628 (PMC10044179; doi:10.1192/bjo.2022.628)
Supplement: Supplementary file 1 [file S2056472422006287sup001.docx]

**Supplementary Material**

Supplementary Figure 1. Flowchart of referral selection

Assessment

n=855,573

Other/no diagnosis

n=740,341

0,341

Diagnosis of depression

n=115,232

Baseline PHQ-9: < 10 or missing

n=19,209

Baseline PHQ-9: > 9

n=96,023

Other/no treatment

n=69,542

Majority CBT or CFD

n=26,481

Equal amounts of two different HIT (treatment ties)

n=322

One majority HIT

n=26,159

Not discharged

n=758

Discharged

n=25,401

Post-treatment PHQ-9 missing

n=2,317

Post-treatment PHQ-9

n=23,084

Previous referrals

n= 1,005

Most recent referral

n=22,079

Pre-2012

n= 2,810

2012-2019

n=19,269

**PHQ-9: Patient Health Questionnaire (9-items); HIT: High-Intensity Therapy*

| Supplementary Table 1. *Summary of data composition for baseline and outcome variables* | | |
| --- | --- | --- |
|  |  |  |
| **Variable** | **Data Type** | **Levels** |
| **Age** | Continuous | - |
| **Gender** | Categorical | *Female* |
|  |  | *Male* |
| **Ethnicity** | Categorical | *White* |
|  |  | White |
|  |  | *Black, Asian, ethnic minority* |
|  |  | Mixed |
|  |  | Asian or Asian British |
|  |  | Black or Black British |
|  |  | Other Ethnic Group |
| **Employment Status** | Categorical | *Employed* |
|  |  | *Not working* |
|  |  | Unemployed and Seeking Work |
|  |  | Students who are undertaking full (at least 16 hours per week) or part-time (less than 16 hours per week) education or training and who are not working or actively seeking work |
|  |  | Long-term sick or disabled, those who are receiving Incapacity Benefit, Income Support or both; or Employment and Support Allowance |
|  |  | Homemaker looking after the family or home and who are not working or actively seeking work |
|  |  | Not receiving benefits and who are not working or actively seeking work |
|  |  | Unpaid voluntary work who are not working or actively seeking work |
|  |  | Retired |
| **Index of Multiple Deprivation** | Continuous | - |
| **Sexual Orientation** | Categorical | *Heterosexual* |
|  |  | *Not heterosexual* |
|  |  | Gay/Lesbian |
|  |  | Bi-sexual |
| **Disability** | Categorical | *No* |
|  |  | *Yes* |
| **Long-Term Health Condition** | Categorical | *No* |
|  |  | *Yes* |
| **Diagnosis** | Categorical | *Depressive episode* |
|  |  | *Recurrent depressive disorder* |
| **Baseline PHQ-9** | Continuous | - |
| **Baseline GAD-7** | Continuous | - |
| **Baseline WSAS** | Continuous | - |
| **Psychotropic Medication** | Categorical | *Yes* |
|  |  | Prescribed and taking |
|  |  | *No* |
|  |  | Prescribed but not taking |
|  |  | Not Prescribed |
| **Referral Source** | Categorical | *Self* |
|  |  | Self |
|  |  | Carer |
|  |  | *Primary Care* |
|  |  | General Medical Practitioner |
|  |  | Health Visitor |
|  |  | Other Primary Health Care |
|  |  | *Other* |
|  |  | Local Authority Services |
|  |  | Employer |
|  |  | Justice System |
|  |  | Child Health |
|  |  | Independent/Voluntary Sector |
|  |  | Acute Secondary Care |
|  |  | Other Mental Health NHS Trust |
|  |  | Internal referrals from Community Mental Health Team (within own NHS Trust) |
|  |  | Internal referrals from Inpatient Service (within own NHS Trust) |
|  |  | Transfer by graduation (within own NHS Trust) |
|  |  | Other |
|  |  | IAPT |
| **Referral Number** | Continuous | - |
| **Low-Intensity Therapy** | Categorical | *No* |
|  |  | 0 low-intensity appointments |
|  |  | Yes |
|  |  | > 0 low-intensity appointments |
| **Post-Treatment PHQ-9** | Continuous | - |

**PHQ-9: Patient Health Questionnaire (9-item); GAD-7: Generalised Anxiety Disorder Scale (7-item); WSAS: Work and Social Adjustment Scale.*

| Supplementary Table 2. *Baseline characteristics stratified by treatment and data split* |  |  |  |  |  |  |
| --- | --- | --- | --- | --- | --- | --- |

|  | | | | |  |  |  |  |  |  |
| --- | --- | --- | --- | --- | --- | --- | --- | --- | --- | --- |
|  |  | *Training Data* | | | | *Testing Data* | | | | *Training & Testing Data* |
|  |  | **Cognitive Behavioural Therapy** | **Counselling for Depression** | **Standardised Mean Difference** | **% Missing** | **Cognitive Behavioural Therapy** | **Counselling for Depression** | **Standardised Mean Difference** | **% Missing** | **Standardised Mean Difference** |
| ***n*** |  | *10,908* | *3544* |  |  | *3636* | *1181* |  |  |  |
| **Age** |  | 39.52 (14.02) | 42.17 (13.68) | 0.192 | 0.0 | 39.79 (14.00) | 42.71 (13.84) | 0.210 | 0.0 | 0.024 |
| **Gender** | Female | 7118 (65.3) | 2502 (70.6) | 0.115 | 0.1 | 2361 (64.9) | 856 (72.5) | 0.163 | 0.1 | 0.005 |
|  | Male | 3790 (34.7) | 1042 (29.4) |  |  | 1275 (35.1) | 325 (27.5) |  |  |  |
| **Ethnicity** | White | 8977 (82.3) | 2549 (71.9) | 0.249 | 4.3 | 3006 (82.7) | 869 (73.6) | 0.221 | 4.7 | 0.017 |
|  | Black, Asian, ethnic minority | 1931 (17.7) | 995 (28.1) |  |  | 630 (17.3) | 312 (26.4) |  |  |  |
| **Employment Status** | Employed | 5827 (53.4) | 2105 (59.4) | 0.121 | 0.8 | 1939 (53.3) | 699 (59.2) | 0.118 | 0.9 | 0.002 |
|  | Not working | 5081 (46.6) | 1439 (40.6) |  |  | 1697 (46.7) | 482 (40.8) |  |  |  |
| **Index of Multiple Deprivation** |  | 21.60 (12.10) | 21.29 (11.01) | 0.027 | 0.4 | 21.40 (12.16) | 21.07 (10.94) | 0.028 | 0.5 | 0.017 |
| **Sexual Orientation** | Heterosexual | 10,350 (94.9) | 3422 (96.6) | 0.083 | 16.5 | 3466 (95.3) | 1128 (95.5) | 0.009 | 15.4 | 0.004 |
|  | Not heterosexual | 558 (5.1) | 122 (3.4) |  |  | 170 (4.7) | 53 (4.5) |  |  |  |
| **Disability** | No | 9328 (85.5) | 3027 (85.4) | 0.003 | 8.3 | 3087 (84.9) | 1007 (85.3) | 0.010 | 8.6 | 0.014 |
|  | Yes | 1580 (14.5) | 517 (14.6) |  |  | 549 (15.1) | 174 (14.7) |  |  |  |
| **Long-Term Health Condition** | No | 7080 (64.9) | 2395 (67.6) | 0.057 | 8.3 | 2368 (65.1) | 759 (64.3) | 0.018 | 8.2 | 0.014 |
|  | Yes | 3828 (35.1) | 1149 (32.4) |  |  | 1268 (34.9) | 422 (35.7) |  |  |  |
| **Diagnosis** | Depressive episode | 7708 (70.7) | 3035 (85.6) | 0.368 | 0.0 | 2580 (71.0) | 1002 (84.8) | 0.339 | 0.0 | 0.001 |
|  | Recurrent depressive disorder | 3200 (29.3) | 509 (14.4) |  |  | 1056 (29.0) | 179 (15.2) |  |  |  |
| **Baseline PHQ-9** |  | 18.53 (4.34) | 17.39 (4.39) | 0.262 | 0.0 | 18.47 (4.40) | 17.45 (4.38) | 0.232 | 0.0 | 0.006 |
| **Baseline GAD-7** |  | 14.51 (4.48) | 13.84 (4.53) | 0.150 | 0.1 | 14.53 (4.50) | 13.68 (4.71) | 0.183 | 0.1 | 0.006 |
| **Baseline WSAS** |  | 23.78 (8.38) | 20.79 (8.77) | 0.349 | 0.1 | 23.73 (8.38) | 21.42 (8.74) | 0.270 | 1.7 | 0.014 |
| **Psychotropic Medication** | Yes | 6655 (61.0) | 1704 (48.1) | 0.262 | 2.9 | 2224 (61.2) | 529 (44.8) | 0.333 | 2.9 | 0.014 |
|  | No | 4253 (39.0) | 1840 (51.9) |  |  | 1412 (38.8) | 652 (55.2) |  |  |  |
| **Referral Source** | Self | 6427 (58.9) | 1545 (43.6) | 0.432 | 0.0 | 2180 (60.0) | 517 (43.8) | 0.433 | 0.0 | 0.018 |
|  | Primary care | 3654 (33.5) | 1895 (53.5) |  |  | 1196 (32.9) | 628 (53.2) |  |  |  |
|  | Other | 827 (7.6) | 104 (2.9) |  |  | 260 (7.2) | 36 (3.0) |  |  |  |
| **Referral Number** |  | 1.86 (1.31) | 1.57 (1.00) | 0.250 | 0.0 | 1.85 (1.29) | 1.55 (0.99) | 0.259 | 0.0 | 0.014 |
| **Low-Intensity Therapy** | No | 7604 (69.7) | 2869 (81.0) | 0.263 | 0.0 | 2565 (70.5) | 969 (82.0) | 0.273 | 0.0 | 0.020 |
|  | Yes | 3304 (30.3) | 675 (19.0) |  |  | 1071 (29.5) | 212 (18.0) |  |  |  |
| **Service** | A | 2803 (25.7) | 292 (8.2) | 1.321 | 0.0 | 938 (25.8) | 126 (10.7) | 1.210 | 0.0 | 0.059 |
|  | *B* | 1522 (14.0) | 7 (0.2) |  |  | 515 (14.2) | 4 (0.3) |  |  |  |
|  | *C* | 904 (8.3) | 805 (22.7) |  |  | 295 (8.1) | 252 (21.3) |  |  |  |
|  | *D* | 1336 (12.2) | 56 (1.6) |  |  | 446 (12.3) | 26 (2.2) |  |  |  |
|  | *E* | 666 (6.1) | 117 (3.3) |  |  | 208 (5.7) | 27 (2.3) |  |  |  |
|  | *F* | 613 (5.6) | 806 (22.7) |  |  | 193 (5.3) | 248 (21.0) |  |  |  |
|  | *G* | 491 (4.5) | 58 (1.6) |  |  | 170 (4.7) | 28 (2.4) |  |  |  |
|  | *H* | 602 (5.5) | 97 (2.7) |  |  | 182 (5.0) | 30 (2.5) |  |  |  |
|  | *I* | 349 (3.2) | 427 (12.0) |  |  | 135 (3.7) | 113 (9.6) |  |  |  |
|  | *J* | 74 (0.7) | 35 (1.0) |  |  | 23 (0.6) | 18 (1.5) |  |  |  |
|  | *K* | 678 (6.2) | 280 (7.9) |  |  | 225 (6.2) | 120 (10.2) |  |  |  |
|  | *L* | 118 (1.1) | 27 (0.8) |  |  | 47 (1.3) | 8 (0.7) |  |  |  |
|  | *M* | 115 (1.1) | 10 (0.3) |  |  | 48 (1.3) | 4 (0.3) |  |  |  |
|  | *N* | 108 (1.0) | 51 (1.4) |  |  | 32 (0.9) | 18 (1.5) |  |  |  |
|  | *O* | 529 (4.8) | 476 (13.4) |  |  | 179 (4.9) | 159 (13.5) |  |  |  |
| **Year** | 2012 | 989 (9.1) | 169 (4.8) | 0.280 | 0.0 | 335 (9.2) | 47 (4.0) | 0.304 | 0.0 | 0.031 |
|  | 2013 | 1415 (13.0) | 342 (9.7) |  |  | 486 (13.4) | 117 (9.9) |  |  |  |
|  | 2014 | 1738 (15.9) | 781 (22.0) |  |  | 541 (14.9) | 270 (22.9) |  |  |  |
|  | 2015 | 2264 (20.8) | 872 (24.6) |  |  | 800 (22.0) | 272 (23.0) |  |  |  |
|  | 2016 | 1552 (14.2) | 514 (14.5) |  |  | 503 (13.8) | 170 (14.4) |  |  |  |
|  | 2017 | 1602 (14.7) | 428 (12.1) |  |  | 524 (14.4) | 156 (13.2) |  |  |  |
|  | 2018 | 1126 (10.3) | 317 (8.9) |  |  | 378 (10.4) | 117 (9.9) |  |  |  |
|  | 2019 | 222 (2.0) | 121 (3.4) |  |  | 69 (1.9) | 32 (2.7) |  |  |  |

**PHQ-9: Patient Health Questionnaire (9-item); GAD-7: Generalised Anxiety Disorder Scale (7-item); WSAS: Work and Social Adjustment Scale. Continuous data are presented as mean (standard deviation) and categorical data are presented as n (%).*

*Table 3. Predictors of post-treatment PHQ-9 score in Cognitive Behavioural Therapy and Counselling for Depression*

|  | | | | |
| --- | --- | --- | --- | --- |
|  | **Beta** | **95% Confidence Interval** | | **p-value** |
| **Age** | -0.04 | -0.05 | -0.03 | <0.001 |
| **Gender (Male)** | 0.11 | -0.15 | 0.36 | 0.400 |
| **Ethnicity (Black, Asian, ethnic minority)** | 0.34 | -0.02 | 0.69 | 0.064 |
| **Employment Status (Not working)** | 1.91 | 1.66 | 2.17 | <0.001 |
| **Index of Multiple Deprivation** | 0.03 | 0.01 | 0.04 | <0.001 |
| **Sexual Orientation (Not heterosexual)** | 0.51 | -0.04 | 1.05 | 0.071 |
| **Disability (Yes)** | 0.68 | 0.31 | 1.05 | <0.001 |
| **Long-Term Health Condition (Yes)** | 0.56 | 0.28 | 0.84 | <0.001 |
| **Diagnosis (Recurrent depressive disorder)** | 0.30 | 0.00 | 0.60 | 0.051 |
| **Baseline PHQ-9** | 0.37 | 0.33 | 0.40 | <0.001 |
| **Baseline GAD-7** | 0.13 | 0.10 | 0.16 | <0.001 |
| **Baseline WSAS** | 0.07 | 0.06 | 0.09 | <0.001 |
| **Psychotropic Medication (No)** | -0.46 | -0.73 | -0.20 | <0.001 |
| **Referral Source (ref: Self)** |  |  |  | <0.001 |
| *Primary Care* | 0.66 | 0.35 | 0.96 |  |
| *Other* | 0.97 | 0.50 | 1.44 |  |
| **Referral Number** | 0.31 | 0.21 | 0.41 | <0.001 |
| **Low-Intensity Therapy (No)** | 0.18 | -0.13 | 0.50 | 0.258 |
| **Service (ref: A)** |  |  |  | <0.001 |
| *B* | 0.50 | 0.03 | 0.98 |  |
| *C* | 1.33 | 0.56 | 2.10 |  |
| *D* | -0.38 | -0.80 | 0.04 |  |
| *E* | -0.80 | -1.34 | -0.26 |  |
| *F* | 0.79 | -0.15 | 1.73 |  |
| *G* | 0.50 | -0.13 | 1.14 |  |
| *H* | -0.71 | -1.29 | -0.14 |  |
| *I* | 0.65 | -0.39 | 1.70 |  |
| *J* | -0.39 | -1.71 | 0.93 |  |
| *K* | 0.45 | -0.16 | 1.05 |  |
| *L* | -1.06 | -2.14 | 0.02 |  |
| *M* | 1.06 | -0.08 | 2.20 |  |
| *N* | 0.47 | -0.71 | 1.64 |  |
| *O* | -0.08 | -0.91 | 0.74 |  |
| **Year (ref: 2015)** |  |  |  | 0.016 |
| *2012* | -0.10 | -0.59 | 0.39 |  |
| *2013* | -0.48 | -0.90 | -0.05 |  |
| *2014* | 0.13 | -0.21 | 0.46 |  |
| *2016* | -0.28 | -0.64 | 0.08 |  |
| *2017* | -0.55 | -0.93 | -0.18 |  |
| *2018* | -0.14 | -0.56 | 0.27 |  |
| *2019* | 0.01 | -0.71 | 0.72 |  |
| **Propensity Score** | 0.45 | -1.65 | 2.54 | 0.676 |

**PHQ-9: Patient Health Questionnaire (9-item); GAD-7: Generalised Anxiety Disorder Scale (7-item); WSAS: Work and Social Adjustment Scale.*
